# Evaluation of nonadherence to treatment among patients with schizophrenia attending psychosocial care centers in the south region of Brazil

**DOI:** 10.1590/2237-6089-2019-0072

**Published:** 2020-10-08

**Authors:** Camila Poletto Viveiros, Camila Rodrigues Tatar, Deivisson Vianna Dantas dos Santos, Sabrina Stefanello, Renato Nisihara

**Affiliations:** 1 Faculdade Evangélica Mackenzie do Paraná CuritibaPR Brazil Faculdade Evangélica Mackenzie do Paraná, Curitiba, PR, Brazil.; 2 Departamento de Saúde Coletiva Universidade Federal do Paraná CuritibaPR Brazil Departamento de Saúde Coletiva, Universidade Federal do Paraná (UFPR), Curitiba, PR, Brazil.; 3 Departamento de Medicina Forense e Psiquiatria UFPR CuritibaPR Brazil Departamento de Medicina Forense e Psiquiatria, UFPR, Curitiba, PR, Brazil.; 4 Departamento de Medicina Universidade Positivo CuritibaPR Brazil Departamento de Medicina, Universidade Positivo, Curitiba, PR, Brazil.

**Keywords:** Schizophrenia, psychosocial care center, clinical profile, epidemiological profile, nonadherence

## Abstract

**Introduction:**

The treatment of schizophrenia aims to reduce symptoms, improve quality of life and promote recovery from debilitating effects. Nonadherence to treatment is related to several factors and may lead to persistence of symptoms and relapse. Worldwide, the rate of nonadherence to treatment in individuals with schizophrenia is around 50%.

**Objectives:**

To compare the clinical profile of nonadherent and adherent patients among individuals diagnosed with schizophrenia receiving treatment at psychosocial care centers in a city in southern Brazil.

**Method:**

The clinical-epidemiological profile of patients with schizophrenia was retrospectively analyzed based on medical records entered into the system between January and December 2016, evaluating data at one-year follow-up.

**Results:**

112 patients were included. The disease was more prevalent in men; mean age was 40.5 years, being lower among men. Most of the sample had a low level of education, was unemployed/retired, did not have children and resided with relatives. The highest rate of diagnosis was among young adults. Psychotic symptoms were most frequently described, and the most commonly prescribed antipsychotic was haloperidol. The nonadherence rate was 15.2%; only one patient required admission to a psychiatric hospital. Among nonadherent patients, the mean time of attendance was 6 months; there were more nonadherent women than men. The most prevalent age range of nonadherence was 41-64 years. Psychosocial and clinical data were similar across the whole sample.

**Conclusion:**

A nonadherence rate of 15.2% was found among individuals receiving treatment for schizophrenia, suggesting that psychosocial care centers were effective in treating and monitoring these patients.

## Introduction

Schizophrenia is a complex chronic mental disorder of idiopathic origin. The most widely accepted explanation for the development of schizophrenia is related to the interaction of multiple factors, including genetic, physical, psychological and environmental factors.^1^ Disorders of the schizophrenia spectrum cause severe mental changes, with characteristic symptoms such as distorted perception and thinking, affection inability and impaired emotional functioning; intellectual impairment may also be present. The condition has a variable evolution, with around 30% of the patients suffering from significant and persistent deterioration of professional, social and affective capacity.^2^ The prevalence of schizophrenia is approximately 1% in Latin America and also specifically Brazil,^3 , 4^ with an incidence of 1 up to 7 new cases per 10,000 inhabitants per year.^5^

The mainstream treatment of schizophrenia aims to achieve three goals: reducing or halting/interrupting symptoms; improving quality of life and functional adaptation; and promoting recovery from debilitating effects.^2^ A better prognosis is related to acceptance of the disorder,^6^ and a central part of treatment adherence is the doctor-patient relationship, where the process of cultivating and establishing rapport with the patient is essential. Nonadherence to drug treatment may lead to relapse in patients in remission and to persistence of symptoms in symptomatic individuals.^7^ The rate of nonadherence to treatment in individuals with schizophrenia is around 50%, causing not only a worsening of the prognosis but also an increase in costs with probably preventable hospitalizations, in addition to being associated with a higher risk of suicide.^8^ Nonadherence may be related to several factors, such as lack of an understanding about the disorder, direct impact of symptoms, social isolation, substance abuse, and increasing fragmentation of mental health services in several countries.^7^ Another relevant factor is the adverse effects of medications in use, such as dysphoria, weight gain and sedation.^9^

In Brazil, the main role of public mental health services such as psychosocial care centers (Centros de Atenção Psicossocial [CAPS]) and psychosocial care nuclei (Núcleo de Atendimento Psicossocial [NAPS]) is to guarantee full, unrestricted access of patients to health care in any level (primary, secondary and tertiary care level), producing new ideas and considerations regarding mental health clinics and the current care model.^10^ These services, created to replace the old hospital-centered model, are intended to change the paradigm in which people with mental disorders are inserted, to rehabilitate them psychosocially, through an expanded multidisciplinary action, taking into account different areas of life – social, personal and family –, therefore aiming for their reintegration into society.^11^ In this sense, the CAPS are structured to provide clinical assistance, organized in each neighborhood.^12^

Few studies have been conducted in Brazil to investigate treatment outcomes in patients diagnosed with schizophrenia. The present study aimed to compare the clinical profile of nonadherent and adherent patients among individuals diagnosed with schizophrenia attending the CAPS.

## Methods

This retrospective study was approved by the ethics committee of Faculdade Evangélica Mackenzie do Paraná (CAAE 58662516.7.0000.0103), and by the City Hall of Curitiba (protocol 114/2016). Data were collected from the clinical records of patients attending the following CAPS in the city of Curitiba: CAPS II Bigorrilho, CAPS III Gate, CAPS III Boa Vista, CAPS III Boqueirão. Clinical records were selected based on the month of entry into the database and on criteria from the F20 spectrum (schizophrenia) of the International Classification of Diseases, 10th revision (ICD-10). All cases receiving assistance between January and December 2016 were included, and each user was studied for the period of one year of follow-up, using the date of entry as base.

The medical records of all patients diagnosed according to the F20 spectrum (schizophrenia), aged over 18 years, which started assistance between January 1 2016 to December 2016 at any CAPS located in Curitiba were included. Patients with a previous diagnosis of neurological diseases were excluded. Analysis of the medical records focused on demographic characteristics (sex, age, marital status, education and profession), psychosocial profile (children, drugs, comorbidities and housing) and clinical data (age range upon diagnosis, family history, symptoms reported at the time of attendance, medications used during the study period and reports of drug side effects). According to their age, patients were divided into young adults (18-40 years), middle age (41-64 years) and the elderly (≥ 65 years). Patients who did not complete the one-year follow-up were considered nonadherent (established previously by the researchers).

The data collected were organized using Microsoft Excel^©^. Statistical analyses were performed using Graph Pad Prism 5.0. Continuous variables were expressed as mean ± standard deviation and compared using the Mann-Whitney test. Categorical variables were expressed as percentages and compared using the chi-square test or Fisher’s exact test, as appropriate. Significance was set at 5%.

## Results

A total of 115 medical records of users diagnosed with schizophrenia were assessed; three records were excluded due to concomitance with other neurological diseases.

The demographic data of the patients assessed are shown in Table 1 . There was a predominance of males (68.7%, 7:1 ratio). Overall, the mean age was 40.5±13.76 years, but men were significantly younger (p ≤ 0.0001). In the group over 65 years there were 5 patients, of whom 3 (60.0%) were men. Most patients had up to 9 years of study (41.1%), and only 8 patients completed up to 16 years of study (7.1%). Among the 112 users, 72 (64.3%) were unemployed or retired. Regarding marital status, 86.6% of the sample were single, divorced or widowed; 77 (68.7%) did not have children and 86 (76.8%) lived with relatives. Only 18 patients (16.0%) lived by themselves.

**Table 1:** Sex, age, age range upon diagnosis, family history, use of alcohol or drugs and comorbidities of patients with schizophrenia (n = 112)

Variable	n (%)
Sex	
Female	35 (31.3)
Male	77 (68.7)
Age (years)	
Young adults (18-40 years)	56 (50.0)
Middle age (41-64 years)	51 (45.5)
Elderly (≥ 65 years)	5 (4.5)
Female mean age	49.2±12.14 years
Male mean age	36.5±12.64 years
Age range upon diagnosis	
Teenager (12-17 years)	2 (1.8)
Young adult (18-40 years)	34 (30.3)
Middle age (41-64 years)	10 (9)
Elderly (≥ 65 years)	2 (1.8)
Not described	63 (56.2)
Median (IQR)	26 (19-42) years
Family history	
Yes	30 (26.8)
No	31 (27.7)
Not described	51 (45.5)
Use of drugs or alcohol	
Yes	36 (32.1)
No	71 (63.4)
Not described	5 (4.5)
Comorbidities	
Systemic arterial hypertension	12 (10.7)
Diabetes mellitus	5 (4.5)
Dyslipidemia	4 (3.6)
Others	18 (16.1)
Without comorbidities	83 (74.1)
Not described	5 (4.5)

IQR = interquartile range.

Psychotic alterations were the most frequently reported symptomatology: 69 users (61.6%) presented hallucinations and 66 (58.9%) had delusions. Among the delusion subtypes, the most prevalent one was the persecutory delusion, which affected 36.6% of the sample. Aggressiveness was present in 33.9% of users and psychomotor agitation in 27.7%.

The full description of the medications used is detailed in Table 2 . Concerning the number of drugs used during the period of one year, the largest fraction of the study population used 1-3 medications, with a median of 3.

**Table 2:** Prescribed medications, number of medications and side effects reported by patients with schizophrenia studied (n = 112)

Medication	n (%)
Antipsychotics	
Haloperidol	55 (49.1)
Risperidone	43 (38.4)
Olanzapine	34 (30.3)
Chlorpromazine	30 (26.8)
Quetiapine	5 (4.5)
Clozapine	5 (4.5)
Levomepromazine	4 (3.6)
Thioridazine	2 (2.8)
Benzodiazepines	
Diazepam	34 (30.3)
Clonazepam	19 (17)
Midazolam	1 (0.9)
Lorazepam	1 (0.9)
Antidepressants	24 (21.4)
Mood stabilizers	23 (20.5)
Biperiden	21 (18.7)
Other	8 (7.1)
No medication	3 (2.7)
Number of medications used	
None	3 (2.7)
1-3	71 (63.4)
4-6	36 (32.1)
≥ 7	2 (1.8)
Side effects	
No complaints	92 (82.1)
Other	10 (8.9)
Sedation	7 (6.2)
Tremors	4 (3.6)
Sialorrhea	3 (2.7)

The treatment outcomes of users with schizophrenia studied here are described in Table 3 . The nonadherence rate was 15.2% (17/112). Among these patients, eight (47.0%) were female; therefore, considering the total of patients studied, there were more nonadherent women than men (p = 0.03). Table 4 shows information about the patients who did not adhere to treatment. In addition, 35.3% had up to 9 years of study, and the same percentage had up to 12 years of study; 41.2% were unemployed and 11.8% retired; 52.9% were single; 58.8% did not have children; and 76.5% lived with their family. The mean age upon diagnosis among nonadherent patients was 42.8 years, and 82.3% had no comorbidities. The most frequent symptoms described in this group were delusions (64.7%), aggressiveness (41.2%), hallucinations (47%), persecutory delusions and auditory hallucinations (both 29.4%). The most used medications were haloperidol (41.2%), chlorpromazine (35.3%) and clonazepam (29.4%). Mood stabilizers were prescribed for 23.5% of the patients, and antidepressants for 17.6% of them. In addition, 88.3% of these patients did not report any drug-related side effects.

**Table 3:** Treatment outcomes among patients with schizophrenia (n = 112)

Outcome	n (%)
Still in the service	63 (56.2)
Transferred to another psychosocial care center	3 (2.7)
Transferred to day hospital	1 (0.9)
Transferred to primary healthcare unit	27 (24.1)
Hospitalized	1 (0.9)
Did not adhere	17 (15.2)

**Table 4:** Follow-up time, sex and age of nonadherent patients (n = 17)

Variable	n (%)
Months of follow-up	
1-6	11 (64.7)
7-11	6 (35.3)
Mean	5.7 months
Sex	
Female	8 (47.1)
Male	9 (52.9)
Total	17 (100.0)
Age	
Young adult (18-40 years)	6 (35.6)
Middle age (41-64 years)	9 (52.9)
Elderly (≥ 65 years)	2 (11.8)
Female mean age	50.2 years
Male mean age	40.5 years

## Discussion

Few studies have described the sociodemographic profile of patients diagnosed with schizophrenia in the Brazilian population.^6 , 8^ These studies are indispensable since the disorder has a chronic course and affects 1% of the population,^5^ with one-third of the affected individuals exhibiting progressive deterioration of professional, social and affective capacity over time.^2^ Nonetheless, the study of treatment nonadherence in patients with schizophrenia is of special importance, since nonadherence to treatment is one of the main determining factors of prognosis, increasing the chances of relapse and frequent hospitalizations.^13^

Data on the prevalence of schizophrenia according to sex differ in the literature^5 , 14^ ; however, some authors have shown a predominance of males in the services evaluated.^15 , 16^ In the present study, the majority of users were male, with a 7:1 ratio. This difference could be explained by specific study characteristics, such as length of assessment and diverse locations of data collection.^17^

Most of the patients in the present sample were of productive age, i.e., between 18-40 years old (mean age: 40.5). Similar results were found in another study^15^ that evaluated the psychiatric population hospitalized in the state of Bahia, where the predominant age range was between 22-50 years. Also, the current study showed that men were affected by the disorder at a younger age when compared to women. Early onset of the disorder in males could be explained by two main hypotheses: the most supported hypothesis points to estrogen as a protective factor in women, while the second etiology for the phenomenon refers to differences in intrauterine development between the genders.^18 - 20^

There was a prevalence of low educational level in the present study, i.e., 41.1% of the patients had up to 9 years of formal education. This finding differs from another study in which the majority of patients with mental disorder being treated at a CAPS in the city of Curitiba had more than 8 years of schooling.^21^

Regarding the professional status of users diagnosed with schizophrenia, the greater part of the patients assessed were retired or unemployed. A study carried out inside a CAPS in the state of Sergipe corroborates these findings: in that study, 75.4% of users with schizophrenia designated themselves as “without profession.”^16^ The demographic characteristics of patients with schizophrenia, with a main impact on the beginning of adult life, hindering their access to the job market, could justify the high prevalence of unemployment.^16^ In addition, the high rate of retirees may be associated with the degree of mental impairment associated with the disorder, which is accompanied by a significant depletion of the user’s work capacity.^2^ The CAPS provide therapeutic support through workshops in a variety of areas that are important in the process of reintegrating users with mental disorders into society, strengthening links and developing or reinforcing skills with the aim of preparing them for the job market and the possibility of income generation.^22^

Most of the patients evaluated were single, divorced or widowed – data that corroborate findings from other studies.^21^ The prevalence of users with children was 30.4%; we did not find reference to this variable for patients with schizophrenia in the literature. As expected, social interactions are worsened in affected patients. In this sense, it is relevant to emphasize that the presence of and connection with family during the treatment of individuals with the disorder show a close relationship with quality of life^23^ and adherence to treatment.^24^ In the sample studied, most users reported living with relatives. Comparable data were not found in the literature, however family engagement in the treatment of these patients should be encouraged.^24^ The greater inclusion of family members enables the development of the patient’s capacities and stimulates social relations.^24^ Approaching the user along with their family is considered necessary and a priority within the treatment project planned by the multidisciplinary teams of the CAPS, with a final goal of engaging relatives in patient care.^22^

The most frequent age range upon diagnosis of the disorder was the young adult range, i.e., between 18-40 years. This finding is consistent with the literature, which describes a similar onset age, at a mean of 18-25 years in men and 25-35 years in women.^5^ As mentioned previously, hypotheses for the later development of the disorder in women could involve estrogen protection and intrauterine development.^18 - 20^

Symptoms often associated with schizophrenia include hallucinations, delusions, thinking and speech disorders, disturbance of emotions and affection, cognitive deficits, and avolition.^25^ Among the symptoms found in the present study, there was a prevalence of psychotic symptoms, such as delusions and hallucinations.

One of the medications most frequently indicated for the treatment of schizophrenia are antipsychotics^6 , 26^ ; in this category, haloperidol stands out, which was used by approximately half of the sample. Less than a quarter of the patients complained about side effects. There is extensive debate in the literature about the side effects of antipsychotics, highlighting metabolic effects such as extrapyramidal symptoms, increased appetite and thirst, sedation, xerostomia, elevated glycemic levels, dyslipidemia and metabolic syndrome.^27 - 29^ Sedation was the effect most commonly observed in our sample. Non-identification of side effects could be explained both by the lack of specific interviewing by a psychiatrist and the patient’s inability to distinguish between symptoms that are inherent to the disorder itself and those related to the medications.

The rate of treatment withdrawal in patients with schizophrenia may reach 50%^8^ and may be even higher than the rates observed for other chronic diseases.^7^ In the present study, a nonadherence rate of only 15.2% was found. This value suggests the effectiveness of the multidisciplinary and integrative model carried out by the CAPS, with high rates of treatment maintenance along one year. This finding could be compared to the effectiveness of CAPS in other areas of mental health, such as a reduction of 14% in the risk of suicide in 2017 in municipalities that offered the service.^30^ These low rates of nonadherence could be explained by the interdisciplinary nature of the work developed at CAPS and also by the model of care adopted, i.e., more focused on the neighborhood when compared to other services. In addition, the CAPS have a greater connection with the health care network in their area of coverage, further reinforcing the link between the user and the service. Also, the CAPS present greater flexibility as care providers, offering a wide range of activities, informal spaces for listening to patients, different care arrangements, preserving the users’ autonomy and freedom.^22^

In the current study, among the nonadherent patients, most had remained in the service for 1-6 months, similar to data found in the literature.^31^ A study that evaluated a population of 6,731 patients diagnosed with schizophrenia in 10 European countries found that, among those who did not adhere to treatment, the majority were men.^32^ With regard to male vs. female nonadherent patients, there were significantly more nonadherent women in the current sample. We did not find similar findings in the literature. The age group with the highest prevalence of nonadherence was the middle age group (41-64 years old), also different from data found in the literature, which reports a higher withdrawal rate in younger patients with mental disorders.^31 , 33 , 34^ Finally, among the nonadherent patients, most were single, which is consistent with other studies.^31^ The marital status of users who abandoned treatment could be related to quality of life, i.e., a lower motivation to adhere to treatment. Single individuals with schizophrenia, of both sexes, are known to have a worse quality of life.^35^

The symptoms presented by patients with schizophrenia could be another factor related to treatment abandonment. Previous work has shown that individuals with negative symptoms present lower adherence rates than those with positive symptoms.^36^ In the present study, nonadherent patients had symptoms such as delusions and aggressiveness, which are considered positive symptoms. Despite the fact that these symptoms are considered positive, the withdrawal observed could be explained by the users’ difficulty interpreting reality and therefore accepting the treatment offered by the service.

Atypical antipsychotics (risperidone, clozapine, olanzapine) are related to a lower rate of nonadherence to treatment in patients with schizophrenia.^8^ These agents may cause serious side effects, such as agranulocytosis, dyslipidemia, among others, even though the usual dosage does not produce significant extrapyramidal symptoms. Conversely, atypical antipsychotics perform better in the presence negative, emotional and cognitive symptoms.^37 , 38^ In this study, the antipsychotics most commonly used among nonadherent patients were typical antipsychotics, namely haloperidol and chlorpromazine. However, most individuals did not report side effects. These data do not agree with the literature, which relates lower rates of adherence mainly to drug side effects. This may also be a reflection of the study method, as not all complaints may have been recorded in the medical chart.

One of the limitations of our study is a population bias, as it relied on a sample of users diagnosed with schizophrenia specifically via the Brazilian Unified Health System. The social and economic characteristics of this population may have affected data such as schooling and housing conditions. Furthermore, the absence of certain data in the medical records may have influenced the profile found in terms of clinical data such as age range upon diagnosis and family history. In addition, the risk of selection and recall bias should have considered. In spite of the limitations, we believe that this study could be reproduced in different regions throughout the country, aiming to obtain a more complete profile of the Brazilian CAPS users with schizophrenia. Altogether, other studies could add information and help in the planning and implementation of public policies capable of improving the health care provided to patients with schizophrenia.

## Conclusions

In the present study, a treatment nonadherence rate of 15.2% was found among patients with schizophrenia, suggesting that the CAPS were effective in treating and monitoring these patients.
